# Knowledge of Toxoplasmosis among Doctors and Nurses Who Provide Prenatal Care in an Endemic Region

**DOI:** 10.1155/2011/750484

**Published:** 2011-05-18

**Authors:** Laura Berriel da Silva, Raquel de Vasconcelos Carvalhaes de Oliveira, Marizete Pereira da Silva, Wendy Fernandes Bueno, Maria Regina Reis Amendoeira, Elizabeth de Souza Neves

**Affiliations:** ^1^Laboratório de Doenças Febris Agudas, Instituto de Pesquisa Clínica Evandro Chagas, Fiocruz, Avenida Brasil 4365, 21040-360 Rio de Janeiro, RJ, Brazil; ^2^Laboratório de Epidemiologia, Instituto de Pesquisa Clínica Evandro Chagas, Fiocruz, 21040-360 Rio de Janeiro, RJ, Brazil; ^3^Vice-Direção de Ensino, Instituto de Pesquisa Clínica Evandro Chagas, Fiocruz, 21040-360 Rio de Janeiro, RJ, Brazil; ^4^Laboratório de Toxoplasmose, Instituto Oswaldo Cruz, Fiocruz, 21045-900 Rio de Janeiro, RJ, Brazil

## Abstract

Congenital toxoplasmosis is a potentially severe infection and its prevention is most often based on serological screening in pregnant women. Many cases could be prevented by simple precautions during pregnancy. Aiming to assess the knowledge about toxoplasmosis among professionals working in antenatal care in a high prevalent region, a questionnaire was administered to 118 obstetric nurses and physicians attending at primary care units and hospitals. The questionnaire was self-completed and included questions on diagnosis, clinical issues, and prevention. Only 44% of total answers were corrected. Lower scores were observed among those with over 10 years of graduation, working in primary care units, and nurses. Errors were mainly observed in questions of prevention and diagnosis. As congenital toxoplasmosis is a mother-to-child (MTC) transmitted disease, early diagnosis and treatment can prevent serious and irreversible fetal damage. Thus, doctors and nurses who provide prenatal care must be appropriately trained on prophylactic, diagnostic, and clinical aspects of toxoplasmosis. The authors suggest that measures should be taken for continuing education regarding toxoplasmosis in pregnancy.

## 1. Introduction

Toxoplasmosis, a zoonosis caused by obligate intracellular protozoan *Toxoplasma gondii* [1, 2], occurs in about one third of world population. Having the cats as definitive hosts, especially the domestic cat, it shows a wide range of intermediate hosts, including humans [3]. In immunocompetent individuals, the infection usually is benign and self-limited. Severe forms are secondary to infection in immunocompromised patients or when acquired during pregnancy, when it may cause congenital disease with severe visual and neurological impairment [4].

Transplacental transmission of *T. gondii* occurs during the period of acute maternal infection [5–7]. The rate of congenital toxoplasmosis with risk for severe fetal varies from 15 to 68%, depending on gestational age; the transmission rate is highest in the later stages of pregnancy [8, 9]. The congenital disease is characterized by a broad spectrum of clinical manifestations with neurological, ophthalmological, and systemic involvement. Although most newborns with congenital infection show no signs of infection at birth, up to 85% of these will develop visual impairment in their lives, and 55% will present neurological disorders [10–12]. The prenatal care, when done properly and from the beginning of the pregnancy, allows early diagnosis of infection, allowing the team more time and resources to treat the fetus [13]. There are few studies addressing the degree of knowledge on toxoplasmosis of the health professionals.

In the few surveys carried out among obstetricians, a deficit in the knowledge about the diagnostic, clinical, and epidemiological aspects of toxoplasmosis was demonstrated, with the inherent risk of inadequate management [14–17]. Nevertheless, similar surveys were not developed in countries with high prevalence of toxoplasmosis as Brazil [18–20], where a previous study observed little knowledge on the subject from pregnant women, in particular regarding the infection prevention [21]. In a previous work, the authors observed that the majority (81.3%) of the women sent to a referral service with a diagnosis of toxoplasmosis were coming from public health units and that for only 16% of them treatment was initiated at the origin unit [22]. This study aims to assess the knowledge about toxoplasmosis by doctors and nurses involved in prenatal care at public health units in Brazil.

## 2. Methods

 We conducted a cross-sectional study of 118 physicians and nurses who provide prenatal care at public health units of a midsize Brazilian city. We chose the city of Juiz de Fora, in Minas Gerais (−21°45′51′′S and 43°21′01′′W), with 500,000 inhabitants and altitude tropical climate. This city was selected because it is a located in a region of high prevalence of *Toxoplasma* infection and offers the following services for the care of pregnant women: basic health units with the Family Health Program (FHP) and three public hospitals. 

All physicians and nurses providing care in all basic units of the Family Health Program (FHP), prenatal clinics, and public hospitals were invited to participate. The study excluded the professionals who did not provide prenatal care last year, who works exclusively in rural areas, and those who did not agree to participate.

Data was collected through an anonymous and self-completed questionnaire, including questions related to the professional profile and nine items about toxoplasmosis equally distributed among diagnosis, clinical issues, and prevention (Table 1).

 EpiData version 3.1 was the software used with double capture and control for data entry. The analysis was performed in the statistic *software Statistical Package for Social Sciences* (SPSS) Version 16.0.

In exploratory analysis of the data, we described the frequencies of qualitative variables and summary measures, such as mean and standard deviation for continuous variables. In continuous variables, we checked the data normality (total number of correct answers in each of the variables: diagnosis, clinical issues, and prevention) by the Kolmogorov-Smirnov test. At the 5% level of significance, the hypothesis of normally distributed data was rejected, which indicated the use of nonparametric tests.

To check for differences in the number of correct answers and the different professional performances (FHP or hospital) was used Mann-Whitney test. *P* values <.05 indicated significant differences, as demonstrated by the comparison of medians (Md) and their respective interquartile ranges (IQRs).

In checking the association between qualitative variables (sex, time from graduation, type of graduation, profession, expertise, previous experience with pregnant women with toxoplasmosis, areas of expertise) and the total number of correct scored (up to four correct answers and over four correct answers) the independence Pearson Chi-square test was used. For tables with low counts (less than five) Fisher's exact test was used. *P* values <.05 indicated significant associations. The cutoff of the total number of correct answers was categorized according to the median of total correct answers.

The study was approved by the Ethics in Research Committee from the Institute of Clinical Research Evandro Chagas (*Instituto de Pesquisa Clínica*:IPEC)-Fiocruz under the protocol 0029.0.009.000-09. All participants signed a consent form.

## 3. Results

Of the 118 professionals who participated in the study, 112 respondents completed the questionnaire (61 physicians and 56 nurses), and six of them did not agree to respond the questionnaire. 

The population consisted mostly of women (80.5%), age 39 ± 9.7, with a degree in public schools (88.2%) and postgraduation *Stricto* and *Lato Sensu*.

Seventy percent (83) of professionals worked at primary FHP and 30.0% in public hospitals. Fifteen percent also worked in private hospitals, 13.7% in private clinics, and 12% in educational and/or research institutions. Fifteen percent of the respondents reported having provided care for pregnant women with toxoplasmosis in the past 12 months.

In the block of questions related to the diagnosis, most professionals said that repeated serology for toxoplasmosis in pregnant women is needed for those who are not immune. The greatest number of correct scores concentrated on clinical issues. Regarding prevention, 97.4% of professionals recognized the cat as the animal that eliminates the parasite in the feces, but 51.7% said the dogs also eliminate oocysts. The largest number of errors was evident in relation to the education of nonimmune pregnant women in regard to not ingesting raw vegetables (Table 1).

The studied population scored 44.4% correct answers on the questions asked. The overall median from the hospital was significantly higher than the FHP's median. Comparing the number of correct answers according to the area of the professional expertise, the professionals who worked in hospitals showed the total number of correct answers on the issues of diagnosis, significantly higher than those who worked in the FHP. Both professionals in hospitals and the FHP had more correct answers to questions about clinical issues and fewer correct answers on prevention (Table 2).

In the comparison according to the professions, the physicians had the highest number of correct answers in the diagnostic and clinical issues. Regarding prevention, no significant differences were found between the two professional categories. The issues of prevention had lower scores both among physicians and nurses (Table 3).

Table 4 presents the results of the analysis of the association of the total number of correct answers classified (up to four or >4 correct answers) with several characteristics of the professionals. Greater number of correct answers was observed in professionals with time of graduation up to 10 years and among physicians.

## 4. Discussion

The Basic Health Units (BHUs) are the main gateway to the Health System in Brazil, and the professionals who work at the Family Health Strategy have a wide work area. Therefore, it is necessary that health professionals have a general education as the proper conduct of health problems depends crucially on the degree of the professional's knowledge. As it is a mother-to-child-(MTC-) transmitted disease, early diagnosis and treatment can prevent serious and irreversible fetal damage [13, 23]. Thus, it is critical that doctors and nurses who provide prenatal care are appropriately trained on the prophylactic, diagnostic, and clinical aspects of the MTC-transmitted diseases. In relation to *T. gondii* infection, its complex life cycle [24], the variable clinical spectrum including mostly oligosymptomatic presentation [4], and no consensus on guidelines contribute to the ignorance or mistaken notions of professionals with regard to this zoonosis.

Regarding the contents of the questionnaire, the block of questions concerning diagnosis, most professionals have responded correctly that it is necessary to repeat serology for toxoplasmosis in pregnant women who are not immune. The periodicity of this screening was not asked as the authors opted for a question of minor complexity. Less than half of professionals recognized low avidity as an indicator of recently acquired infection, and an even smaller proportion still knew that IgM anti-*T. gondii* may remain positive for more than a year.

The greatest number of correct answers concentrated on clinical issues, although the responses showed a misunderstanding between the period of greatest transmissibility and the period of increased severity of congenital injuries [25, 26]. About prevention, although 97.4% of professionals recognize the cat as the animal that eliminates the parasite in feces, professionals attributed this role, which is unique to *Felidae* [27, 28], to other animals as well, especially dogs (51.7%) and pigeons (21.6%). The largest number of errors was evident in relation to the orientation of non-immune pregnant women as to not eating raw vegetables. As oocyst has a major importance in the epidemiology of toxoplasmosis, and taking into account water as an important font of infection and oocyst resistance to chlorine and acetic acid, the authors recommend that eating raw salads and unpeeled fruits should be avoided in nonimmune Toxoplasma pregnant women in Brazil [18, 29–31]. 

The fact that the overall median number of correct answers and diagnosis from professionals working in hospitals were higher than those of the FHP may in part be explained by the fact that professionals working in hospitals have more training in high-risk prenatal care. However, a higher number of correct questions of prevention among professionals FHP were expected, since the basic health unit has as one of its main missions educational activities aimed at preventing health problems [29]. Likewise, the median number of correct answers among physicians higher than that of nurses is expected in the diagnosis and clinical issues, as these issues are the object of focus in medical schools than in schools of nursing. However, the basic knowledge of nurses in issues related to prevention is inconsistent with the role of nurses as health educators [30]. Eleven (15.3%) respondents reported having provided care to pregnant women with toxoplasmosis in the last 12 months, two times the percentage already considered high in a study conducted in the United States [14], implying that a considerable proportion of doctors and nurses encounter over their lives with at least one case of a pregnant woman with toxoplasmosis.

The greatest number of correct answers among the professionals with less training time is consistent with the literature that indicates an inverse correlation between knowledge and years of professional practice, justifying the need for recertification exams in some countries [31, 32]. Continued education seems especially useful when targeted to specific groups and disciplines [33] like our target population. The low ratio observed (44.4%) of correct answers on questions about global toxoplasmosis reinforces the need for action and continuing education programs on this zoonosis.

Finally, the authors suggest that similar surveys shall be conducted in other endemic regions and with larger populations. The establishment of a cutoff for the number of correct answers can be a useful tool for a possible intervention, providing support to actions for continuing education about pregnancy and congenital toxoplasmosis for these occupational categories.

## Figures and Tables

**Table 1:** Frequency distribution of responses about toxoplasmosis applied to doctors and nurses who provide prenatal care at public health units, Juiz de Fora Brazil, 2009.

Questions	Answers	%
*Diagnosis*		
(1) Recent infection, active. May be indicated by		
High antitoxoplasma IgG avidity.	35	30.4
Low antitoxoplasma IgG avidity*.	41	35.7
IgG avidity does not relate to time of infection.	21	18.3
I do not know.	18	15.7
(2) Regarding IgM class antitoxoplasma antibodies we may state		
They indicate recent infection and disappear in < 6months.	60	52.2
They indicate recent infection and may persist for over a year*.	33	28.7
They indicate past infection and do not relate to infection time.	16	13.9
I do not know.	6	5.2
(3) A pregnant woman presenting negative *Toxoplasma* serology needs to repeat the test?		
Yes*.	94	81.7
No.	10	8.7
Only if she presents fever, asthenia and enlarged lymph nodes.	10	8.7
I do not know	1	0.9

*Clinical*		
(1) Concerning toxoplasmosis clinical manifestations in pregnant women		
Most cases present fever, asthenia and enlarged lymph nodes.	16	13.9
Its main manifestations are arthritis and rash.	0	0
Compromises primarily the central nervous system.	8	7
Most cases are asymptomatic*.	85	73.9
I do not know.	6	5.2
(2) In which gestational quarter (trimester) congenital infection risk is the greater?		
First quarter.	58	50.4
Second quarter.	6	5.2
Third quarter*.	27	23.5
It is even all along pregnancy.	22	19.1
I do not know.	2	1.7
(3) In which gestational quarter (trimester) toxoplasmosis severity is the greater?		
First quarter*.	75	65.8
Second quarter.	5	4.4
Third quarter.	14	12.3
It is even all along pregnancy.	13	11.4
I do not know.	7	6.1

*Prevention *		
(1) The animals that eliminate the *Toxoplasma* in the environment through their feces are		
Dogs.	60	51.7
Cats*.	113	97.4
Pigeons.	25	21.6
Pigs.	9	7.8
Lambs.	14	12.1
Ox/cows.	9	7.8
I do not know.	1	0.9
(2) Regarding raw vegetables, preventive measures for non immune pregnant women are		
Use chlorine to clean raw vegetables.	66	56.9
Use acetic acid to clean raw vegetables.	21	18.1
Raw vegetables should be eliminated from the diet*.	6	5.2
Raw vegetables are not important in toxoplasmosis epidemic	11	9.5
I do not know.	12	10.3
(3) Please indicate the raw meat that should be avoided in non immune pregnant women:		
Ox/cow*.	84	72.4
Pig*.	70	60.3
Lamb*.	38	32.8
Fish.	7	6
Crustacean	6	5.2
I do not know	19	16.4

*Correct answers

**Table 2:** Comparison of the number of correct answers about toxoplasmosis among professionals in the Family Health Program (FHP) and at the hospital, who provide prenatal care at public health units, Juiz de Fora, Brazil, 2009.

Variables	FHP		Hospital		*P* value*
	Median	IQR	Median	IQR	
Total number of correct answers (*N* = 108)	3.0	2.0–5.0	4.0	3.0–6.0	**.028**
Number of correct answers on diagnosis (*N* = 110)	1.0	1.0–3.0	2.0	1.0–3.0	**.029**
Number of correct answers on clinical issues (*N* = 109)	2.0	1.0–2.0	2.0	1.0–3.0	.315
Number of correct answers on prevention (*N* = 111)	0.0	0.0–1.0	1.0	0.0–1.0	.134

*P value <.05

**Table 3:** Comparison of the number of correct answers about toxoplasmosis among professionals in the Family Health Program (FHP) and at the hospital, who provide prenatal care at public health units, Juiz de Fora, Brazil, 2009.

Variable	Physicians		Nurses		*P* value*
	Median	IQR	Median	IQR	
Total number of correct answers (*N* = 108)	4.0	3.0–6.0	3.0	2.0–4.0	**<**.001
Number of correct answers on diagnosis (*N* = 110)	2.0	1.0–2.0	1.0	0.0–2.0	.001
Number of correct answers on clinical issues (*N* = 109)	2.0	1.0–3.0	1.0	1.0–2.0	**<**.001
Number of correct answers on prevention (*N* = 111)	1.0	0.0–1.0	0.0	0.0–1.0	.275

*P value < 0.05

**Table 4:** Distribution of variables in the profile of professionals who provide prenatal care at public health units, according to the level of knowledge about toxoplasmosis, Juiz de Fora, Brazil, 2009.

Variable	<4 correct answers		≥4 correct answers		*P* value*
	*n*	(%)	*n*	(%)	
Sex (*N* = 113)					
Male	15	19.5	13	36.1	.056
Female	62	80.5	23	63.9	
Time from graduation					
<10 years	26	33.8	20	55.6	**.035**
11– 20 years	21	27.3	10	27.8	
≥21 years	30	39	6	16.7	
Type of educational institution					
Public university	67	88.2	30	83.3	.556
Private university	9	11.8	6	16.7	
Profession					
Physician	33	42.9	26	72.2	**.004**
Nurse	44	57.1	10	27.8	
Specialization					
Yes	69	89.6	31	86.1	.752
No	8	10.4	5	13.9	
Has ever provided care to a pregnant woman with toxoplasmosis					
Yes	11	15.3	11	31.4	.052
No	61	84.7	24	68.6	
